# Deciphering the Origin of the 2012 Cholera Epidemic in Guinea by Integrating Epidemiological and Molecular Analyses

**DOI:** 10.1371/journal.pntd.0002898

**Published:** 2014-06-05

**Authors:** Stanislas Rebaudet, Martin A. Mengel, Lamine Koivogui, Sandra Moore, Ankur Mutreja, Yacouba Kande, Ousmane Yattara, Véronique Sarr Keita, Berthe-Marie Njanpop-Lafourcade, Pierre-Edouard Fournier, Eric Garnotel, Sakoba Keita, Renaud Piarroux

**Affiliations:** 1 Aix-Marseille Université, UMD 3, Marseille, France; 2 Agence de Medecine Preventive (AMP), Paris, France; 3 Institut National de Santé Publique (INSP), Conakry, Republic of Guinea; 4 Wellcome Trust Sanger Institute, Hinxton, Cambridge, United Kingdom; 5 Division Prévention et Lutte contre la Maladie (DPLM), Ministère de la Santé Publique et de l'Hygiène Publique, Conakry, Republic of Guinea; 6 Aix-Marseille Université, Faculté de Médecine, Marseille, France; 7 Hôpital d'Instruction des Armées (HIA) Alphonse Laveran, Marseille, France; Massachusetts General Hospital, United States of America

## Abstract

Cholera is typically considered endemic in West Africa, especially in the Republic of Guinea. However, a three-year lull period was observed from 2009 to 2011, before a new epidemic struck the country in 2012, which was officially responsible for 7,350 suspected cases and 133 deaths. To determine whether cholera re-emerged from the aquatic environment or was rather imported due to human migration, a comprehensive epidemiological and molecular survey was conducted. A spatiotemporal analysis of the national case databases established Kaback Island, located off the southern coast of Guinea, as the initial focus of the epidemic in early February. According to the field investigations, the index case was found to be a fisherman who had recently arrived from a coastal district of neighboring Sierra Leone, where a cholera outbreak had recently occurred. MLVA-based genotype mapping of 38 clinical *Vibrio cholerae* O1 El Tor isolates sampled throughout the epidemic demonstrated a progressive genetic diversification of the strains from a single genotype isolated on Kaback Island in February, which correlated with spatial epidemic spread. Whole-genome sequencing characterized this strain as an “atypical” El Tor variant. Furthermore, genome-wide SNP-based phylogeny analysis grouped the Guinean strain into a new clade of the third wave of the seventh pandemic, distinct from previously analyzed African strains and directly related to a Bangladeshi isolate. Overall, these results highly suggest that the Guinean 2012 epidemic was caused by a *V. cholerae* clone that was likely imported from Sierra Leone by an infected individual. These results indicate the importance of promoting the cross-border identification and surveillance of mobile and vulnerable populations, including fishermen, to prevent, detect and control future epidemics in the region. Comprehensive epidemiological investigations should be expanded to better understand cholera dynamics and improve disease control strategies throughout the African continent.

## Author Summary

Cholera is a potentially deadly diarrheic disease caused by the toxin-secreting bacterium *Vibrio cholerae*. In many poor countries, this prototypical waterborne disease is considered endemic and linked to the climate-driven proliferation of environmental reservoirs of the pathogen. Although such a statement implies radical public health consequences, it has never been proven in Africa. The present study aimed to elucidate the origin of the cholera epidemic that struck the Republic of Guinea in 2012 following a three-year lull period. This investigation integrated a spatiotemporal analysis of the national case databases, field investigations and thorough genetic analyses of 38 clinical bacterial isolates sampled throughout the Guinean epidemic. The Guinean *V. cholerae* DNA sequence results were aligned and compared with the sequences of nearly 200 strains isolated throughout the world over the past 60 years. Overall, these results suggest that the 2012 cholera epidemic strain was likely imported from Sierra Leone to Guinea by traveling fishermen. The emergence of cholera epidemics due to human-driven activity may be widespread throughout Africa. This highlights the importance of transborder collaborative public health strategies targeting highly mobile and high-risk populations. Similar integrated studies should be conducted in other countries impacted by the disease to better understand the spread of recent epidemics and thus better intercept future outbreaks.

## Introduction

Cholera is generally considered endemic in West Africa [1], especially in countries such as Nigeria, Benin, Togo, Ghana, Liberia and the Republic of Guinea [2]. In 2004–2008, Guinea was struck by a succession of regional cholera outbreaks responsible for 17,638 reported cases and 786 deaths [3]. In 2009, the country established an early cholera alert system including cholera microbiological surveillance to quickly detect emerging epidemics [4]. However, the following years in Guinea were marked by a lull in cholera transmission until new cases were reported between February and April 2012 in several maritime prefectures spanning 200 km [5]. Between April and June, a reactive oral cholera vaccination campaign was implemented by the Guinean Ministry of Health and Médecins Sans Frontières (Doctors Without Borders) in two prefectures, Forecariah and Boffa [6]. However, during the rainy season in July and August, the epidemic exploded in the capital Conakry and then spread to inland areas. By the time the end of the epidemic was declared in December 2012, 7,350 cases and 133 deaths had been officially reported to the World Health Organization (WHO), from 11 out of 34 prefectures [7].

To provide a scientific foundation for the control and prevention of future outbreaks, it is critical to understand the origin of cholera epidemics in coastal areas, which has remained subject to debate. In Peru and Bangladesh, a similar near simultaneous appearance of cholera at different locales along coastal or estuarine areas has been considered a key argument in favor of the “cholera paradigm” [8]. According to this general model for cholera transmission, coastal waters in these regions represent reservoirs of multiclonal epidemic-provoking *Vibrio cholerae* strains whose growth is directly associated with plankton blooms driven by climatic and environmental conditions [8]–[12]. Conversely, whole-genome-based phylogenetic analyses of Peruvian and other South American isolates from the 1990s have found that the strains form a clonal and independent lineage within the seventh pandemic [13], [14]. Such molecular approaches have recently highlighted the function of human-to-human transmission of the disease [12], which could be the main driver of clonal outbreak diffusion, even along coastal areas.

To assess whether the 2012 Guinean cholera epidemic was caused by local environment-to-human transmission or was rather initiated by the human-driven importation of a single toxigenic clone we used a multidisciplinary approach involving spatiotemporal analyses, field investigations and several complementary *V. cholerae* genotyping methods.

## Materials and Methods

### Cholera cases and deaths, rainfall, population and geographical data

The Republic of Guinea spans 245,857 km^2^ and is administratively divided into 33 prefectures plus the capital Conakry. In 2012, the country had an estimated population of 12 million inhabitants. At that time, the Guinean national health surveillance system prospectively reported all suspected cholera cases based on the WHO definition of the disease [15]. Each Prefectural Health Directorate (DPS – *Direction Préfectorale de la Santé*) tallied new cases recorded at the various health structures of the prefecture on a weekly basis. Aggregated morbidity and mortality cholera data were then transmitted to the Directorate of Prevention and Disease Control (DPLM – *Direction de la Prévention et de la Lutte contre la Maladie*), which compiled the information in a national database of 7,350 cases. DPLM also retrospectively compiled a line list of 6,568 patients, which included the date of consultation and geographical origin down to the village level and was anonymized prior to analysis. To limit notification bias, both databases were subsequently compared and merged, which enabled the retrieval of 393 additional cases. The use of these data for epidemiological, research and publication purposes was approved by the Guinean Ministry of Health (*Ministère de la Santé Publique et de l'Hygiène Publique*). Daily-accumulated rainfall data were obtained from satellite estimates (TMPA-RT 3B42RT derived) provided by the National Aeronautics and Space Administration (available at: http://disc2.nascom.nasa.gov/Giovanni/tovas/realtime.3B42RT_daily.2.shtml). As most cases were recorded in Maritime Guinea and, to a lesser extent, Middle Guinea, daily rainfall data were averaged on the position 9.00N-12.00N/15.00-11.75W, which excluded the eastern two-thirds of the country where precipitation levels were lower and much fewer cholera cases were reported. Population estimates for 2012 were obtained from the Guinean Expanded Program for Immunization at both the prefectural and sub-prefectural levels. Their estimates were based on the general population census of 1996 considering prefecture-specific annual population growth rates, which were provided by the Guinean Statistics National Institute (INS – *Institut National de la Statistique de Guinée*) and ranged from 0.71% to 6.51%.

### Field investigations

Field investigations of index cases and local conditions that supported cholera emergence and transmission were prospectively conducted in affected areas throughout the epidemic by epidemiologists of the Guinean Health Ministry and the country team of the African Cholera Surveillance Network (Africhol; http://www.africhol.org) to organize the public health response. They included basic interviews among affected communities identified by the hospital- and community-based surveillance system (including rumors) and followed routine procedures of the Integrated Disease Surveillance and Response System of the Guinean Ministry of Health. Retrospective field investigations were also conducted in August and September 2012 mainly to review the register books of treatment facilities, but also to interview local health authorities and staff regarding the 2012 outbreak as well as to observe ecological, social, water and sanitation conditions in affected areas.

### Sampling of clinical *V. cholerae* isolates and DNA extraction

With the support of the Africhol Consortium and following standard procedures [16], the reference laboratory of the Public Health National Institute (INSP – *Institut National de Santé Publique*) tested 236 clinical samples positive for *V. cholerae* O1 throughout the duration of the 2012 epidemic, out of which 212 isolates were prospectively stored in a biobank created for that purpose. In September 2012, 50 of these isolates were selected for genotyping, subcultured and then transported in glycerol tubes at room temperature to Marseille, France. Isolates were selected in a manner in which the samples were temporally and spatially representative of outbreak diffusion during the first 8 months of the epidemic and included early and later isolates from all 7 prefectures available in the biobank. Upon arrival in Marseille, the strains were recultivated on non-selective trypticase soy agar (TSA) medium (Difco Laboratories/BD) for 24 hours at 37°C. Suspected *V. cholerae* colonies were identified via Gram-staining, oxidase reaction and agglutination assessment with *V. cholerae* O1 polyvalent antisera (Bio-Rad). For DNA extraction, an aliquot of cultured cells was suspended in 500 µL deionized water, incubated for 10 min at 100°C and centrifuged for 10 min at 1500× g. The pellet was then resuspended in 250 µL deionized water and incubated for 5 min at 100°C. The supernatant (containing DNA) was subsequently stored at −20°C. DNA was directly extracted from the glycerol transport tubes for the isolates that failed to grow upon culture.

### MLVA-based genotyping of sampled strains

Genotyping of the *V. cholerae* strains was performed via MLVA (Multiple Loci VNTR (Variable Number Tandem Repeat) Analysis) of 6 VNTRs (Table 1), including 4 previously described assays [17], [18] and 2 assays specifically designed for this study to improve the discriminating power of the analysis. The novel VNTR assays were designed based on the reference strain El Tor N16961 (GenBank accession numbers AE003852.1 and AE003853.1) using Perfect Microsatellite Repeat Finder webserver (currently unavailable). Specific primer pairs were subsequently designed using the Primer3 program (http://simgene.com/Primer3) (Table 1). Fluorescent-labeled primers were purchased from Applied Biosystems.

**Table 1:** Characteristics and primer sequences of the 6 genotyped VNTRs.

Locus name	Repeated pattern	Chr.^1^	Position^2^	Primer sequence (5′→3′)	Ref
VC1	AACAGA	1	137106	fw: CGGATACTCAAACGCAGGAT	[17], [18]
				rv: 6FAM*-CTTTCGGTCGGTTTCTCTTG	
VC4	TGCTGT	2	187759	fw: TGTTTGAGAGCTCGCCTCTT	[17], [18]
				rv: PET*-TCATCAAGATGCACGACACA	
VC5	GATAATCCA	1	1915539	fw: AGTGGGCACAGAGTGTCAAA	[17], [18]
				rv: VIC*-AATTGGCCGCTAACTGAGTG	
VC9	GACCCTA	1	467111	fw: CGTTAGCATCGAAACTGCTG	[17], [18]
				rv: NED*-AGAAAACAATCGCCTGCTTG	
VCLAV6	ACCAGA	2	303939	fw: NED*-GCCTCCTCAGAAGTTGAGAATC	Present study
				rv: CCGATGAACTCTCTGAACTGG	
VCLAV8	TTGTCGA	1	532253	fw: VIC*-CTCGCTTAAGTTGCCTTACCC	Present study
				rv: GCGAACCAGACGTACTTTCAG	

1 Chr.: chromosome.

2 Based on the reference strain El Tor N16961 (GenBank accession numbers: AE003852.1 and AE003853.1).

PCR thermal cycling conditions for all assays: 95°C for 5 min; followed by 30 cycles of 95°C for 30 sec, 58°C for 30 sec and 72°C for 45 sec; 72°C for 5 min.

For each PCR assay, DNA amplification was carried out by mixing 0.375 µL of each primer (20 µM), 1 X LightCycler 480 Probes Master (Roche Diagnostics) and approximately 100 ng of template DNA in a total volume of 30 µL. PCR was performed using a LightCycler 480 System (Roche Diagnostics) with the thermal cycling conditions described in Table 1. PCR amplicons were subsequently verified via agarose gel (2%) electrophoresis.

VNTR PCR product size was determined via capillary electrophoresis. Aliquots of the PCR products were first diluted 1∶100 in sterile water, which was further diluted 1∶100 in a solution containing 25 µL Hi-Di Formamide 3500 Dx Series (Applied Biosystems) and 0.5 µL GeneScan 500 LIZ Size Standard (Applied Biosystems). The fluorescent end-labeled amplicons were analyzed using an ABI PRISM 310 Genetic Analyzer (Applied Biosystems) with POP-7 Polymer (Applied Biosystems). Finally, amplicon size was determined using GeneMapper v.3.0 software (Applied Biosystems).

### Whole-genome sequencing

To better characterize the *V. cholerae* strains responsible for the epidemic, whole-genome sequencing was performed on a strain isolated at the onset of the epidemic (strain G298_Guinea) using a GS FLX+ System (454 Life Science, a Roche company). The DNA sequence was assembled using Newbler, from GS *De novo* Assembler (http://454.com/products/analysis-software/index.asp).

To perform a phylogenetic assessment of the core *V. cholerae* genome based on genome-wide SNPs (single nucleotide polymorphisms), strain G298_Guinea DNA was re-sequenced using a HiSeq Illumina System (Illumina).

### Statistical and analytical methods

For the spatiotemporal description of the epidemic, rainfall data were aggregated weekly and graphically represented in parallel with cholera morbidity. Cholera attack rates were calculated and mapped, by prefecture and sub-prefecture, for various time periods using shapefiles of administrative divisions obtained from the HealthMapper application (WHO, Geneva, Switzerland) and Quantum GIS v1.8.0 (QGIS Geographic Information System, Open Source Geospatial Foundation Project, available at: http://qgis.osgeo.org).

MLVA-based genotypes were compared at each of the 6 VNTR loci. Genetic relatedness between the strains was first assessed using eBURSTv3 (http://eburst.mlst.net/), which aims to identify the founding genotype. A simple network of all possible links between genotypes was also assembled using Gephi.0.8.1 beta software (https://gephi.org/). Molecular epidemiology analyses were completed via the sequential mapping of each genotype by month at the prefecture level.

After the first whole-genome sequence was obtained with the GS FLX+ System, proteins were predicted using Prodigal software (http://prodigal.ornl.gov/). Data was then annotated employing the GenBank database (http://www.ncbi.nlm.nih.gov/genbank) and the Clusters of Orthologous Groups database using BLASTP with an E-value of 10^−5^. Allelic polymorphism of the cholera toxin B subunit and other virulence factors was characterized by comparing the obtained sequence with the genome description of *V. cholerae* strains available in GenBank and recent literature.

For phylogenetic analyses, the paired-end read data obtained with the HiSeq Illumina System and sequence data from 198 previously sequenced strains available in the NCBI SRA database were mapped to the reference N16961 El Tor strain (NCBI accession numbers AE003852 and AE003853) using SMALT software (http://www.sanger.ac.uk/resources/software/smalt). A whole-genome alignment was obtained for each strain in this analysis, and SNPs were called using the approach described by Harris *et al.* [19]. The reads that did not map to the N16961 genome were filtered out during SNP calling, and any SNP with a quality score less than 30 was excluded. A true SNP was only called if there were at least 75% of the reads at any heterogeneously mapped ambiguous sites. High-density SNP clusters indicating possible recombination sites were excluded using the methodology previously described by Croucher *et al.* [20]. Maximum Likelihood phylogenetic trees were estimated using the default settings of RAxML v0.7.4 [21] based on all the SNPs called in the manner explained above. M66 (accession numbers CP001233 and CP001234), a pre-seventh pandemic strain, was used to root the final phylogenetic tree of the seventh pandemic strains [13]. FigTree (http://tree.bio.ed.ac.uk/software/figtree/) was used to visualize and order the nodes of the phylogenetic tree.

## Results

### Spatiotemporal epidemic progression

**Figure 1:**
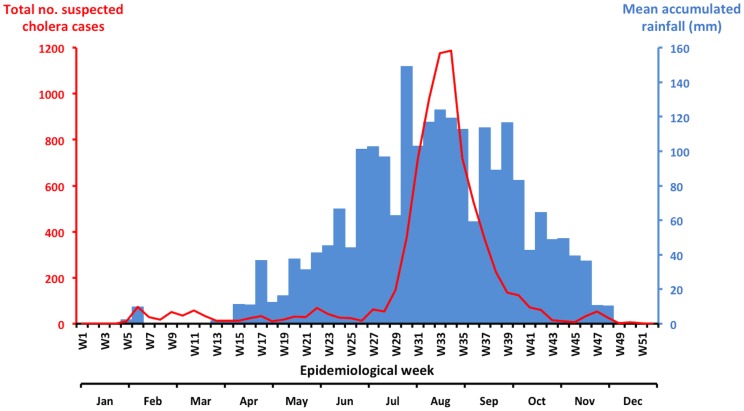
Evolution of the weekly cholera cases and rainfall in Guinea in 2012. Accumulated rainfall data for the most affected areas of the country (Maritime and Middle Guinea) were obtained from satellite estimates (TMPA-RT 3B42RT derived), which was averaged on the position 9.00N-12.00N/15.00-11.75W and is available at: http://disc2.nascom.nasa.gov/Giovanni/tovas/realtime.3B42RT_daily.2.shtml. The blue bars indicate the weekly rainfall levels, and the red line indicates the number of suspected cholera cases per week.

Taking into account both the national database and patient line list, this epidemic was responsible for an estimated 7,743 suspected cases (global attack rate: 6.3 cases/10,000 inhabitants) and 138 deaths (case fatality ratio: 1.8%). The initial case was reported on February 2, 2012 (epidemiological week 5) in the midst of the dry season (Figure 1). The weekly number of new cases remained below 100 until July. The epidemic then peaked in August, 5 months after the onset of the rainy season, with nearly 1,188 new cases recorded during week 34. Cholera incidence began to markedly decline in September. The final case was recorded on December 11, 2012, and the Minister of Health officially declared the end of the epidemic on February 6, 2013.

**Figure 2:**
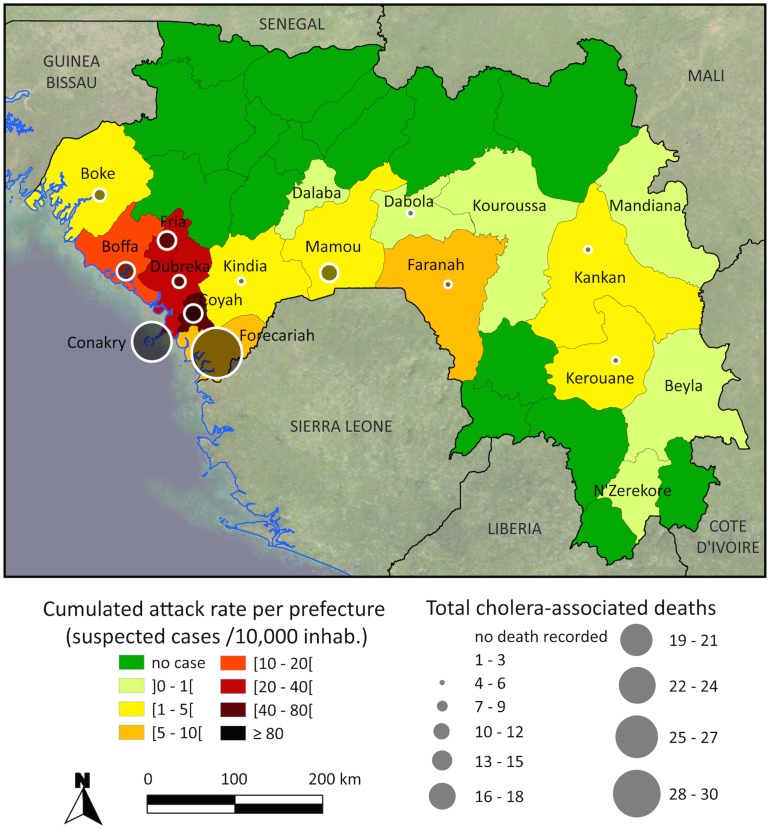
Cumulated cholera attack rates and deaths per prefecture during the 2012 Guinean epidemic.

Overall, the capital of Conakry reported 4,642 cases (25.9 cases/10,000 inhab.), which represented more than half of the national case total, but only 24 deaths (case fatality ratio: 0.5%). Moreover, 2,178 additional patients were located in the 5 other prefectures that border the Atlantic Ocean, with the highest attack rate observed in Coyah (55.1 cases/10,000 inhab.) (Figure 2). Twelve other prefectures were also affected, including distant inland prefectures such as Kerouane (Figure 2).

**Figure 3:**
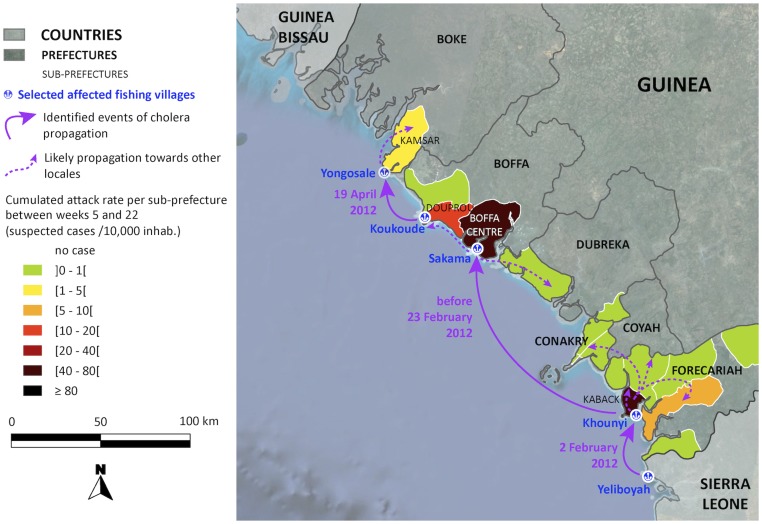
Cholera in Maritime Guinea between February and May 2012. The map illustrates the early propagation of the outbreak along the coast and the cumulated attack rate per sub-prefecture. Village positions are available on the Index Mundi website (http://www.indexmundi.com/zp/gv/).

The initial cholera cases in Guinea emerged on February 2, 2012 on Kaback Island (Prefecture of Forecariah) (Figure 3), which is located in a remote mangrove zone close to the border with Sierra Leone, where an epidemic of acute diarrhea and vomiting had been reported in January. The Guinean index case was a fisherman who had just traveled by boat from Sierra Leone (a village on Yeliboyah Island, Kambia District) and arrived in the fishing village of Khounyi, on a land strip of the southern tip of Kaback Island. During the first month of the epidemic, this small village, which lacked safe water and improved sanitation facilities, recorded over 100 cases and represented the most affected community in the prefecture.

The cholera epidemic then progressively diffused northwestward along the Guinean coast, striking the prefectures of Boffa on February 23 and Boke on April 22 (Figure 3). As observed in Forecariah Prefecture, the initial cases in Boffa and Boke were also reported in fishing camps, namely Sakama and Yongosale, respectively. At each of these lowland fishing locales, the index case was a fisherman who had recently returned from an already affected area (i.e., travelling from Kaback to Sakama and from Koukoude (Boffa prefecture, Douprou sub-prefecture) to Yongosale). Concomitant with the expansion of the epidemic along the coast, cholera had also begun to spread inland. However, the inland prefectures were not significantly affected until the onset of the rainy season. Likewise, although Conakry is situated on a peninsula between the early affected regions of Kaback and Boffa, cholera did not strike the capital until a month after the inception of the rainy season. The first case in Conakry was officially recorded on May 29, who appeared to be a merchant returning from the Kaback market. Conakry subsequently acted as an amplifier of epidemic spread, especially towards the interior portions of the country, where several identified index cases were found to be drivers, merchants or students recently returning from the capital.

### MLVA-based genotyping and strain relatedness analysis

**Figure 4:**
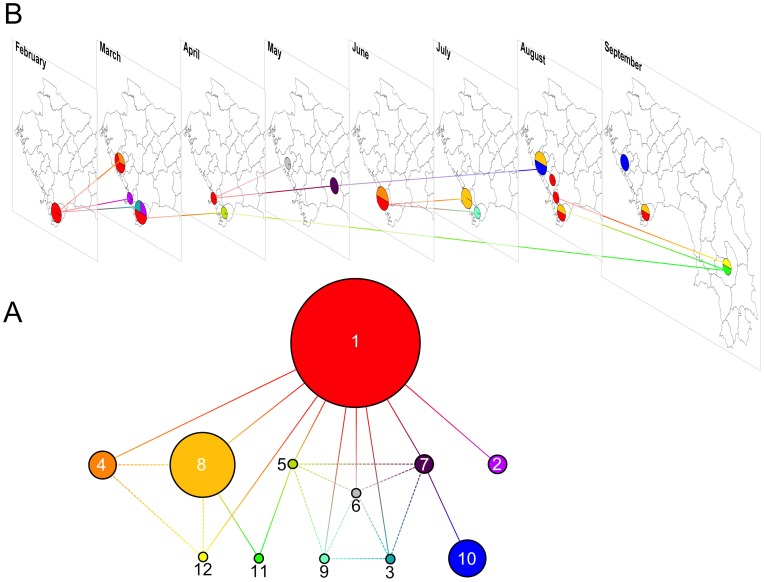
MLVA-based genotypes and relatedness of 38 clinical *V. cholerae* strains isolated in Guinea in 2012. (A) Network of *V. cholerae* strain relatedness based on MLVA genotype. Following the genotype analysis of 38 *V. cholerae* strains using 6 different microsatellite loci, the strains were grouped according to the resulting MLVA genotype profiles. Each colored circle corresponds to a different genotype. The numbers indicate the sequential order when the first strain of the corresponding genotype was isolated. Circle diameter is relatively proportional to the number of isolates represented by each genotype (e.g., 14 strains displayed genotype #1, 7 isolates displayed genotype #8 and 1 strain displayed genotype #3). Each segment corresponds to a single mutation at 1 of the 6 assessed VNTRs. Bold segments represent primary and likely relatedness links between genotypes, while the dashed segments represent secondary and less likely genetic relationships. Genotype #1 was identified as the founder genotype using the eBURST algorithm. (B) Spatiotemporal repartition of genotyped *V. cholerae* strains. Prefecture-level maps of Guinea are displayed for each month from February to September 2012. The genotype color code described in Figure 4A was applied to spatially and temporarily localize the isolated strains. Therefore, the pie charts reflect the month and prefecture of strain isolation (represented by strain genotype) as well as the relative proportion of each genotype among them. Segments between different months spatially and temporarily illustrate the genetic relatedness displayed in Figure 4A. Only primary and sequentially earliest links between genotypes are represented.

Fourteen samples out of 50 were not positive by culture and 2 additional samples were heavily contaminated. However, direct DNA extraction from transport tubes was successful for 4 culture-negative isolates. Genotype analysis with the 6-VNTR panel was thus performed on 38 *V. cholerae* isolates. All strains displayed constant results for the VC1, VC5 and LAV8 assays, while the VC4, VC9 and LAV6 assays revealed 4, 3 and 6 allelic variants, respectively. Based on the MLVA results, the strains were grouped into 12 different genotypic profiles, all of which were very closely related (Figure 4). All strains seemed to have arisen from genotype #1, which was identified as the founder genotype using the eBURST algorithm. Genotype #1 represented the earliest genotype isolated during the 2012 epidemic (on Kaback Island in February 2012) as well as the most frequent genotype identified (Figure 4). Subsequent diversification of this clone occurred via 1 or 2 mutational events during its propagation across the country (Figure 4).

### Whole-genome sequencing of the *V. cholerae* founder clone

**Figure 5:**
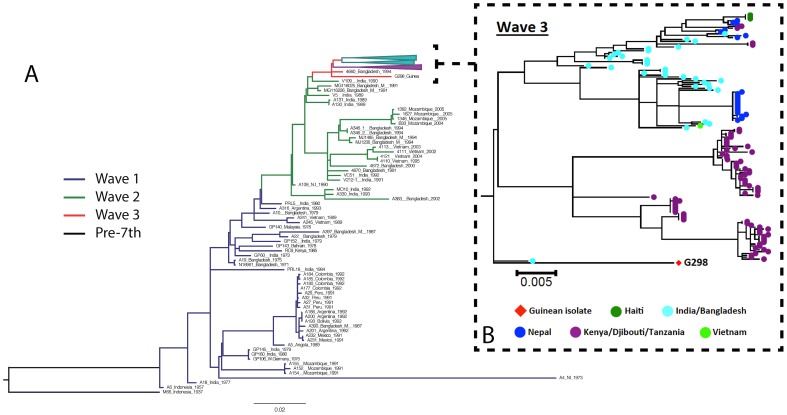
(A) Maximum likelihood phylogenetic tree of the seventh pandemic lineage of *V. cholerae* based on the SNP differences across the whole core genome and including a strain isolated during the onset of the Guinean 2012 outbreak. The pre-seventh pandemic isolate M66 was used as an outgroup to root the tree. Blue, green and red branches represent waves 1, 2 and 3, respectively. Purple and sky blue clade lineages represent the Kenyan clade and two South Asian clades within the third wave, respectively. Scale is provided as the number of substitutions per variable site. (B) Greater resolution of wave 3 of the seventh pandemic, in which the Guinean strain clustered distinctly from the two South Asian clades and the dominant Kenyan clade. Guinean isolate G298 is represented by the square while each colored circle indicates a spatially different isolate (as shown in the key). Scale is provided as the number of substitutions per variable site.

The genome of a genotype #1 strain isolated on February 28, 2012 in Kaback was examined via whole-genome sequencing. The cluster composition of the virulence genes displayed one “hybrid” CTX*ϕ* prophage on chromosome 1 but no RS1 fragment. Sequence results showed that this “hybrid” CTX*ϕ* harbors a majority of El Tor allele genes (e.g., *zot*, *ace* and *cep*) with a classical *ctxB* gene (encoding the B subunit of the cholera toxin) and a classical *rstR* gene. Strain phylogeny based on genome-wide SNP analysis situated this Guinean “atypical” El Tor variant within a new clade of the third and most recent wave of the seventh pandemic (Figure 5). This strain was thus distinct from both strains isolated in Mozambique in 2004–2005 (second wave) and strains isolated between 2005 and 2010 in Eastern Africa (i.e., the Kenyan clade within the third wave, indicated in purple on Figure 5). The Guinean 2012 strain was also found to be clearly separated from two South Asian clades (indicated in sky blue on Figure 5), which includes the Haitian clone. The closest relative of the Guinean strain was a strain isolated in 1994 in Bangladesh.

## Discussion

While tracking the origin of the 2012 Guinean cholera epidemic, this multidisciplinary study demonstrates the monoclonal nature of the epidemic, as clinical *V. cholerae* strains exhibited a progressive genetic diversification that paralleled outbreak diffusion from Kaback Island. Molecular results confirmed the epidemiological findings, as the single ancestral and most abundant genotype was the sole *V. cholerae* strain isolated during the onset of the epidemic in February, at the initial focus of Kaback. According to field investigations, the index case was a fisherman arriving from a nearby cholera-affected district of Sierra Leone. Cholera then bounced along the Guinean coast, likely carried by other infected fishermen, before exploding during the rainy season in the capital Conakry and subsequently spreading inland. This clone was found to be an “atypical” El Tor variant of *V. cholerae*, as determined via whole-genome sequencing. Furthermore, this Guinean strain phylogenetically grouped into a new clade of the third wave of the current pandemic, and the closest known relative was a strain isolated in Bangladesh in 1994. This study represents the first such molecular analysis of a cholera epidemic conducted in West Africa.

Overall, these results strongly suggest that cholera spread along the coast of Guinea due to human-driven diffusion of the bacterium. According to the molecular analyses, this epidemic was caused by a single clone, which rapidly evolved in parallel with the spatiotemporal spread of the epidemic. A few weeks after identification of the founder clone in Kaback, the same genotype was identified in new outbreak foci further along the coast, where it was likely transported by infected traveling fishermen. Likewise, isolates characterized by descendant genotypes were found to have spread across the country throughout the year, with strains of the most distant genotypes primarily identified in distant prefectures, such as Kerouane, several months later (e.g., August and September). Such genotype analysis has rarely been conducted to assess cholera epidemic diffusion from the onset. However, similar genetic diversification from an initial *V. cholerae* clone has been recently observed throughout the current epidemic in Haiti [22], where the human-associated importation of cholera is largely undoubted [23], [24]. Furthermore, the diffusion of cholera by traveling fishermen has already been documented in West Africa. For example, the arrival of the seventh cholera pandemic in Ghana in 1971 was linked to the repatriation of a man who had succumbed to the disease while fishing in the waters of Togo, Liberia and Guinea [25].

Conversely, had the 2012 cholera epidemic originated from a local aquatic reservoir of proliferating vibrios, the diversity of *V. cholerae* strains found in the environment would have resulted in the early identification of several distinct clones [8], [26]. Therefore, the emergence of a unique *V. cholerae* genotype in clinical samples isolated on Kaback Island in February does not correlate with environment-to-human transmission of the disease. Furthermore, this period was not characterized by the wet and warm climatic conditions that are considered to be a favorable to *V. cholerae* proliferation in water bodies [8]–[12]. Finally, a recent review addressing cholera epidemics in African coastal areas has indicated that no perennial environmental reservoir of toxigenic *V. cholerae* O1 has yet been identified in West Africa, which may be attributed to the lack of appropriate studies [27].

The epidemiological data rather suggest that cholera was imported to Guinea from Sierra Leone. Indeed, Kaback is situated less than 30 km away from this neighboring country. Nearby districts of Sierra Leone, including Kambia and Port Loko, were already affected by the disease in early January 2012 [28]. Furthermore, the index case identified in Kaback was a travelling fisherman who had just arrived from a fishing village in Kambia. Unlike Guinea, where an efficient early alert system [4] enabled the detection, report, investigation, laboratory-confirmation and official declaration of the outbreak within 8 days after the appearance of the first cholera case observed in the past 3 years, health authorities in Sierra Leone did not perform similar investigations. Thus, the origin of this cholera epidemic in Sierra Leone remains unclear, although possible importation events by fishermen travelling from Liberia and Ghana have been reported [29].

Finally, according to whole-genome sequence analysis, this epidemic was caused by an “atypical” El Tor variant of *V. cholerae* O1, a type of strain that harbors both El Tor biotype genetic elements and the Classical biotype *ctxB* gene [30]. Such “atypical” El Tor strains initially emerged in Asia in 1991 and were first detected on the African continent in 2004 [31]. This may also present major public health implications as these strains have been suggested to be associated with more severe clinical symptoms compared with conventional El Tor strains [32], [33]. Furthermore, genome-wide SNP-based phylogeny analysis grouped the Guinean 2012 clone into a recent clade within the third wave of the seventh pandemic. Several studies have shown that this monophyletic radiation is largely distinct from the vast diversity of *V. cholerae* environmental strains [14], [34], which suggests that cholera epidemics are clonal and caused by a specific subset of related *V. cholerae* strains often spread via human-to-human transmission [14], [35].

Nevertheless, to confirm the origin of the *V. cholerae* clone responsible for this epidemic, it would have been ideal to analyze pre-epidemic environmental isolates as well as isolates from previous epidemics in Guinea, isolates from Sierra Leone and strains from other countries the region. However, earlier Guinean isolates were not stored and we did not have access to strains from Sierra Leone. Furthermore, no study of environmental *V. cholerae* strains had previously been performed in the region.

In conclusion, by tracking the origin of the 2012 cholera epidemic in the Republic of Guinea, this study identified fishermen as cholera victims and vectors during the early phase of epidemic propagation. Improving water and sanitation infrastructures, implementing enhanced hygiene education programs and targeting oral cholera vaccination campaigns in high-risk coastal areas could thus benefit these vulnerable populations and prevent the spread of future cholera outbreaks. The likely Sierra Leonean origin of this Guinean epidemic highlights the importance of encouraging transborder collaboration in the surveillance and control of highly mobile populations and main communication routes so as to rapidly identify emerging foci and organize coordinated targeted responses. These results also support the implementation of biobanks dedicated to prospective clinical and environmental *V. cholerae* isolates, to perform molecular epidemiological analyses, which have become essential to interpret field investigation data. Such an integrated approach would provide valuable insights concerning cholera in other African regions, where the key determinants of all too frequent epidemics still remain poorly understood and prevention or control strategies are not always accurately oriented.
